# Multimodal AI-driven analysis in breast and lung cancer: insights from the OPTIMA prototyping workshop

**DOI:** 10.1186/s12919-026-00370-8

**Published:** 2026-04-02

**Authors:** Maryam Abdollahyan, Emanuela Gadaleta, Lewis G. E. James, Graeme J. Thorn, Javier Cuadrado Corz, Vivek Singh, Manuel Hettich, Lisa Schneider, Oscar Maiques, Ayman Hijazy, Asieh Golozar, Juan Gómez Rivas, Philip Cornford, Rossella Nicolleti, Thomas Abbott, Eng Hooi Tan, Danielle Newby, Giorgos Papanastasiou, Michael Bussmann, Bertrand De Meulder, Claude Chelala, the PIONEER consortium and the OPTIMA consortium

**Affiliations:** 1 Centre for Cancer Biomarkers and Biotherapeutics, Barts Cancer Institute, Queen Mary University of London, London, UK; 2 Ludwig Maximilian University of Munich, Munich, Germany; 3 R&D Machine Learning Research, Bayer AG Pharmaceuticals Division, Berlin, Germany; 4 Odysseus Data Services, an EPAM Company, New York, NY USA; 5 Department of Urology, Hospital Clinico San Carlos, Madrid, Spain; 6 Liverpool University Hospitals NHS Foundation Trust, Liverpool, UK; 7 Unit of Urological Robotic Surgery and Renal Transplantation, Careggi Hospital, Florence, Italy; 8 European Association of Urology Guidelines Office, Arnhem, The Netherlands; 9 Centre for Statistics in Medicine, Nuffield Department of Orthopaedics, Rheumatology and Musculoskeletal Sciences, University of Oxford, Oxford, UK; 10 Artificial Intelligence Data and Analytics, Pfizer Inc, New York, NY USA; 11 Helmholtz-Zentrum Dresden-Rossendorf, Dresden, Germany; 12 BDM Consulting, Larajasse, France

**Keywords:** Breast cancer, Lung cancer, Artificial Intelligence, Optimal treatment

## Abstract

This paper reports on insights from the OPTIMA (Optimal Treatment for Patients with Solid Tumours in Europe Through Artificial Intelligence) prototyping workshop held in Berlin from November 6 to November 8, 2024. Through integrated analysis of clinical, genomic, imaging and pathology data, we addressed the following key challenges in breast and lung cancer management: utility of comprehensive genomic profiling in metastatic breast cancer settings; relevance of tumor heterogeneity for predicting treatment response; development of less invasive technologies for assessing tumor biology; and treatment outcomes in early stages of small cell lung cancer. Our findings demonstrate the potential of computational analysis using multiple data modalities to identify cancer molecular subtypes and enhance treatment selection and monitoring while highlighting important areas for future development to achieve the research objectives of the OPTIMA consortium.

## Background

Breast cancer remains one of the most prevalent malignancies worldwide, with over 2.3 million new cases diagnosed annually [1]. In the European Union (EU) alone, breast cancer accounts for approximately 29% of all female cancers, with significant variations in mortality rates across member states [2]. Despite advances in early detection and treatment strategies, complexity of breast cancer biology continues to present substantial challenges for optimal patient care, particularly in metastatic settings.

Lung cancer is one of the most common and deadly cancers worldwide. In the EU, approximately 470,000 new cases of lung cancer are recorded annually, accounting for 11.9% of all new cancer diagnoses [3]. In terms of mortality, lung cancer was the leading cause of cancer-related deaths in the EU, responsible for about 20.4% of all cancer [3]. Lung cancer is broadly classified into two main types: non-small cell lung cancer (NSCLC), which accounts for about 85% of cases, and small cell lung cancer (SCLC), which is more aggressive but less common.

The OPTIMA consortium is a groundbreaking initiative that brings together leading European institutions to revolutionize cancer treatment through Artificial Intelligence (AI). Here we present an overview of the Consortium's latest prototyping workshop (held in Berlin from November 6 to November 8, 2024) to streamline computational workflows addressing key challenges in breast and lung cancer care using multimodal data analytics. Through extensive stakeholder engagement and systematic review, the OPTIMA consortium identified several high-priority breast and lung cancer research questions. Among them, four key questions emerged as main focuses for the workshop:

- Q1: The potential of comprehensive genomic profiling in metastatic breast cancer patients to improve outcomes compared to standard therapy, particularly regarding survival, adverse events and quality of life [4, 5].
- Q2: The role of tumor heterogeneity in predicting treatment response in metastatic settings, acknowledging the complex interplay between different tumor cell populations and their impact on therapeutic efficacy [6].
- Q3: The development and validation of less invasive technologies for assessing tumor biology and dynamics, aiming to match or exceed the predictive capabilities of traditional invasive biopsies for treatment planning.
- Q4: The analysis of which therapy sequence results in the best survival outcome for patients with stage I-II SCLC.

While fully addressing these questions presents significant data and computational challenges, this paper reports on the OPTIMA prototyping workshop's efforts to tackle some of the challenges through the development of workflows that address these questions using different breast and lung cancer data modalities. For breast cancer, we present our initial approach to integrating clinical, genomic and imaging data, and demonstrate how computational analysis using multiple data modalities can enhance our understanding of tumor biology, potentially leading to more personalized and effective therapeutic strategies. For lung cancer, we present a workflow to phenotype lung cancer across several data sources using distributed analytics.

## Workflows

### Session 1: extracting information from radiology reports

Presenter: Maryam Abdollahyan.

#### Data sources and preprocessing

This session focused on the unstructured free text information found within electronic health records (EHRs). Multimodal data from the Breast Cancer Now Biobank (BCNB) Barts Cancer Institute (BCI) site were used [7]. Clinical data were mapped to the Observational Medical Outcome Partnership (OMOP) Common Data Model (CDM) [8, 9] and are linked to EHRs from Barts Health NHS Trust (BH). We selected a small cohort of breast cancer patients for whom data from both BCNB and BH, including free text radiology reports, are available over a period of minimum five years – when the risk of breast cancer recurrence is highest – or until death, though it is possible that some reports for these patients were missing (e.g., if the patient moved to another hospital under a different NHS Trust or private healthcare). Only the patients for whom data have been audited by at least two data officers were included in the cohort.

#### Methods

The focus of this session was to leverage Natural Language Processing (NLP) for extracting insights from unstructured EHRs, specifically radiology free text reports, to enable the stratification of breast cancer patients. This stratification, informed by combining these NLP-derived insights with other data such as biobank clinical data, aims to identify groups with distinct characteristics, their risk of disease progression or potential treatment response. Specifically, we demonstrated an example of using (classic) NLP techniques to capture the site of breast cancer metastasis. The workflow was implemented in Python, and Pandas [10] and NLTK [11] libraries were employed for data manipulation and NLP, respectively.

Information such as whether the patient had metastasis (and, if they did, the details including site of metastasis) is found in the OMOP-mapped BCNB data. This information served as ground truth for validating the output of NLP techniques. We retrieved all the radiology reports for these patients from BH EHRs. The reports were filtered based on date to exclude the ones for procedures that were performed prior to the patient’s primary diagnosis. Additionally, the reports were filtered based on modality (mammogram, CT, MRI and ultrasound scans etc.) to exclude the ones that are unlikely to contain breast cancer-related information (e.g., dental X-rays).

Reports typically contain information that is added by the EHR system and is present as structured data. These values (e.g., procedure datetime, modality and reporter name) were identified and removed. Reporters (here, consultant radiologists) often use custom headings (e.g., ‘Clinical Indication’, ‘Findings’ and ‘Conclusion’) to organize the content of their reports into sections. These sections do not adhere to a standard format, i.e., they do not appear in a particular order or hierarchy; features such as capitalization of headings and delimiters were not used consistently; and in some cases, multiple headings were merged into one, separated by a conjunction or special character. We used a rule-based approach to identify the various sections within each report – This required fixing sentence boundary issues. Focusing on one or more sections, instead of the entire report, could improve the performance of clinical NLP tasks. In the demo, all sections were analyzed.

We normalized the reports: shortened words (e.g., acronyms and clipped words such as ‘mets’) were expanded; text was converted to lowercase; special characters were replaced; text was tokenized and lemmatized; stop words were removed; and negation was handled using a look-ahead strategy. To detect mentions of metastatic breast cancer and metastasis site, we mapped the extracted keywords to a standard vocabulary. For simplicity and to eliminate the overhead of installing and configuring additional resources due to time constraints of this session, WordNet (which is a part of the NLTK corpus) was used. For instance, keywords that matched the Synset ‘body part’ based on their common hypernym were flagged as candidates for metastasis site. Furthermore, we generated the *n*-grams (here, sequences of *n* consecutive lemmas) for each sentence in the reports. Next, we searched for bigrams that consist of keywords indicating possible metastasis event and site – Negation was taken into account and effects of increasing the value of *n* were assessed. Finally, the results were validated by comparing the output to labels generated from the OMOP-mapped BCNB data.

#### Results

Analysis of radiology reports demonstrated the potential of NLP techniques to automate the extraction of various information relating to clinical attributes such as metastasis site that could supplement other data and aid clinicians in their decision-making processes.

### Session 2: integrating clinical, genomic and transcriptomic data for patient benefit

Presenters: Emanuela Gadaleta, Lewis GE James and Graeme J Thorn.

#### Data sources and preprocessing

The analysis incorporated three key types of data from The Cancer Genome Atlas Breast Invasive Carcinoma (TCGA-BRCA) cohort [12]: clinical, genomic and transcriptomic. The clinical data demographics and tumor characteristics were linked to the associated genomic and transcriptomic data outputs. The Mutation Annotation Format (MAF) [13] file contained detailed information on somatic variants such as genomic location, reference and altered alleles, variant classification and potential functional impacts. The transcriptomic data consisted of a gene expression matrix. In addition, workshop participants were provided with a curated pharmacogenomic file, derived from pharmacogenomic databases [14, 15], that comprised of drug targets and predicted treatment responses linked to specific mutations.

#### Methods

**Fig. 1:**
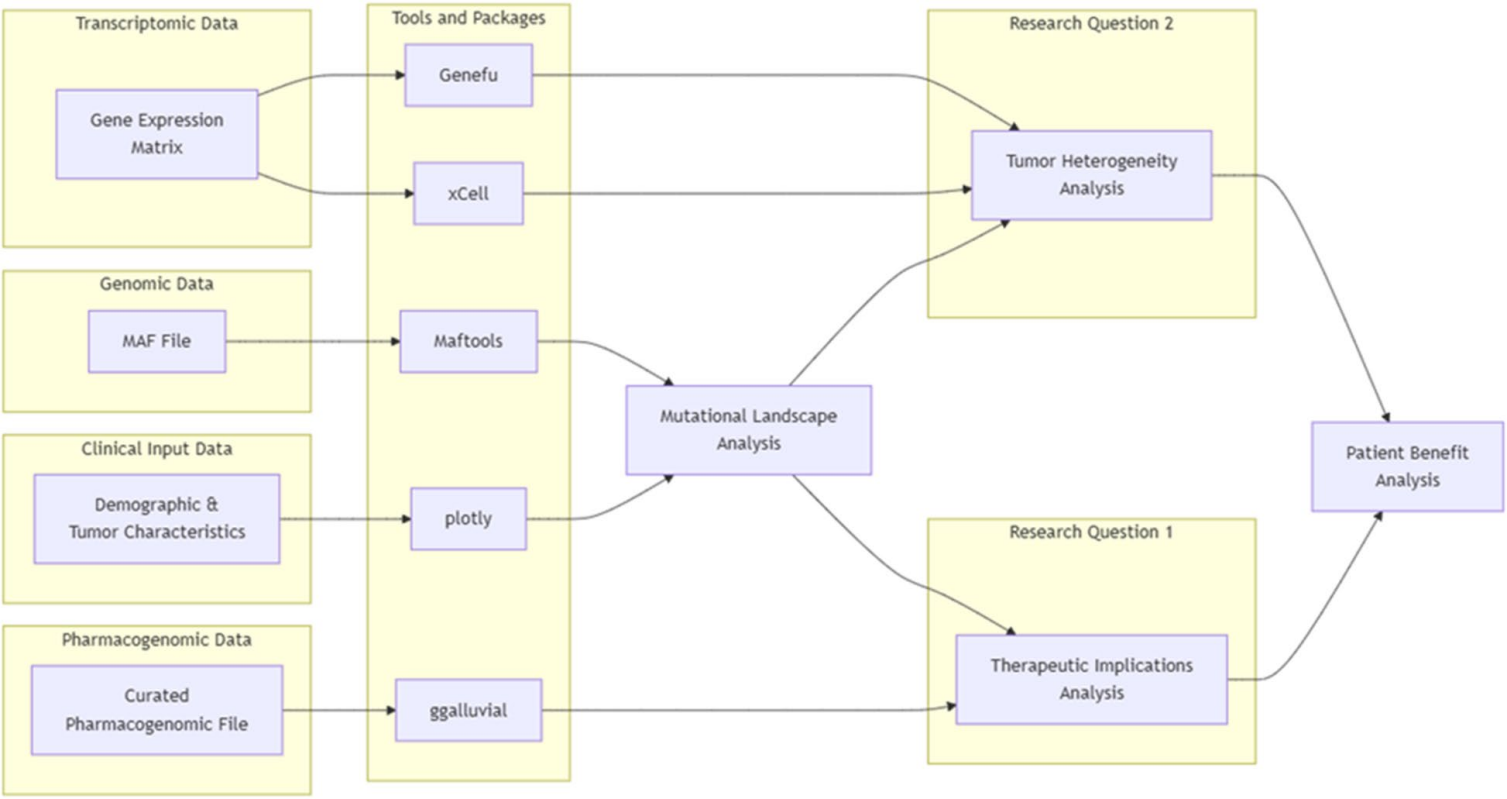
Overview of the pipeline integrating clinical, genomic and transcriptomic data for characterizing the TCGA-BRCA cohort

The pipeline integrated genomic, transcriptomic and pharmacogenomic findings to characterize the mutational landscape of the TCGA-BRCA cohort, focusing on tumor heterogeneity and therapeutic implications for patient benefit. All analyses were conducted in R using the plotly [16], Maftools [17], ggalluvial [18], Genefu [19] and xCell [20] packages (Fig. 1).

#### Results

Demographics were overlaid with tumor characteristics to provide a summarized view of the clinical data. We provided an example illustrating that Black patients presented around 4 years earlier than their White counterparts in the same cohort.

The clinical and genomic data were integrated to display the mutational landscape of the cohort. This highlighted TP53 and PIK3CA as key players in breast cancer progression, with mutation frequencies of 32% and 34%, respectively. We demonstrated how patients can be stratified into clinically relevant groups based on their genomic profile using PIK3CA [21] as an example. The presentation focused on three PIK3CA hotspots – H1047R, E545K and E542K – with important therapeutic implications [22]. We then provided examples of these implications in estrogen receptor (ER) positive and human epidermal growth factor receptor 2 (HER2) positive breast cancer. Further pharmacogenomic evaluations were conducted using the top mutated genes and a curated file to highlight potential drug targets alongside predicted treatment responses.

For analysis of the clinical and transcriptomic data, samples were classified into different molecular subtypes and analyzed further to estimate the abundance of various cell types within the tumor microenvironment. Significant variations in immune infiltration across molecular subtypes were reported. Notably, basal and HER2-enriched subtypes showed higher immune scores compared to luminal subtypes, suggesting differential susceptibility to immunotherapy approaches. These findings were supported by spatial analysis of tissue components through digital pathology of matched images from The Cancer Imaging Archive (TCIA) [23], which revealed distinct patterns of immune cell distribution within the microenvironment (Session 4).

The final part of this session focused on discussing the benefits and challenges of applying profiling, classification and stratification methods to liquid biopsies.

### Session 3: deep learning for prediction of breast cancer malignancy and subtype in mammograms

Presenters: Vivek Singh and Manuel Hettich.

#### Data sources and preprocessing

Mammograms in the Chinese Mammography Database (CMMD) dataset [24] from The Cancer Imaging Archive (TCIA) were acquired using standardized imaging protocols to ensure high-quality images. Each mammogram includes multiple views such as craniocaudal (CC) and mediolateral oblique (MLO). Preprocessing steps, crucial for optimizing model performance, included.

- Resizing: images were resized to match the input requirements of the deep learning (DL) architecture, increasing computational efficiency.
- Intensity normalization: pixel intensities were normalized to ensure consistency across the dataset and reduce input variance.
- Noise reduction: Gaussian filtering was applied to suppress noise and enhance diagnostically relevant features, leading to more effective feature extraction during training.

#### Methods

##### Detection of abnormal regions

A You Only Look Once version 9 (YOLOv9) [25] detector, chosen for its real-time object detection capabilities and optimized for speed and accuracy, was trained on the CMMD dataset to localize regions of interest (ROIs) that potentially contain abnormalities. Focusing on these ROIs reduced computational overhead and improved classification specificity, optimizing resource utilization.

##### Classification pipeline

The identified ROIs were then processed by a classification pipeline performing two tasks:

1. Binary classification: differentiating between benign and malignant abnormalities
2. Molecular subtype classification: predicting four subtypes for malignant cases (Luminal A, Luminal B, HER2-enriched and Triple-Negative)

A ResNet-50 [26] model pretrained on ImageNet [27] was used for feature extraction and classification. Pretraining initializes the model with optimized weights, accelerating convergence during subsequent training. Data augmentation techniques, including rotations, flipping (horizontal and vertical) and scaling/cropping, were employed to enhance model robustness and address potential data limitations, expanding the training dataset and improving generalization.

The models were trained for 100 epochs using mini-batch Stochastic Gradient Descent (SGD). SGD was chosen for its efficiency in navigating high-dimensional parameter spaces. A carefully tuned learning rate was employed to increase convergence speed and avoid oscillations. Early stopping, based on validation loss, was implemented to prevent overfitting and identify the optimal point in the training process. Model checkpoints saved the best-performing weights, ensuring retrieval of the most optimized model. We used the learning rate of 0.0001 and batch size of 32.

##### Performance evaluation

Model performance was evaluated using.

- Accuracy: overall prediction correctness
- Precision: proportion of true positive predictions among all positive predictions
- Recall (sensitivity): ability to correctly identify positive cases
- Area Under the Receiver Operating Characteristic Curve (AUROC): overall discriminatory power

These metrics assessed performance for both binary and multiclass classification, providing a comprehensive evaluation of the optimization process.

##### Visualization and feature analysis

Gradient-weighted Class Activation Mapping (Grad-CAM) [28] provides a visual explanation for a particular prediction generated by a DL network. It produces a heatmap overlaid on the original mammogram, highlighting pixels and areas that significantly impacted the model's decision-making. The primary objective of Grad-CAM is to validate that the model's rationale corresponds with clinical expertise. If the model categorizes a mammogram as malignant, the heatmap should accurately identify the lesion or tumor that a radiologist would recognize. In contrast, for a benign categorization, the heatmap must highlight the distinct characteristics of the benign finding. This approach serves as an essential validation step: if the heatmap focuses on an insignificant artifact or a random area of the mammogram, it would indicate that the model has acquired inaccurate patterns. Grad-CAM clearly shows the model's focus, offering convincing case-specific evidence that increases a clinician’s confidence in the output before advancing to further stages such as expert radiologist assessment or treatment planning.

t-Distributed Stochastic Neighbor Embedding (t-SNE) [29] is a visualization approach that explains a model's overall analysis of the data. A t-SNE plot for a successful stage 1 model shows two distinct and well-separated clusters: one representing benign cases and the other representing malignant cases. For a stage 2 model, the malignant cluster shows four additional subclusters representing the Luminal A, Luminal B, HER2-enriched and Triple-Negative molecular subtypes. The distinct separation of these clusters validates the discriminative efficacy of the characteristics obtained by the ResNet-50 model. This demonstrates that the model has effectively discerned fundamental patterns robust enough to differentiate not only between benign and malignant conditions but also among complex molecular subtypes, instilling confidence in its ability for precise classification and subtype categorization tasks.

#### Results

**Fig. 2:**
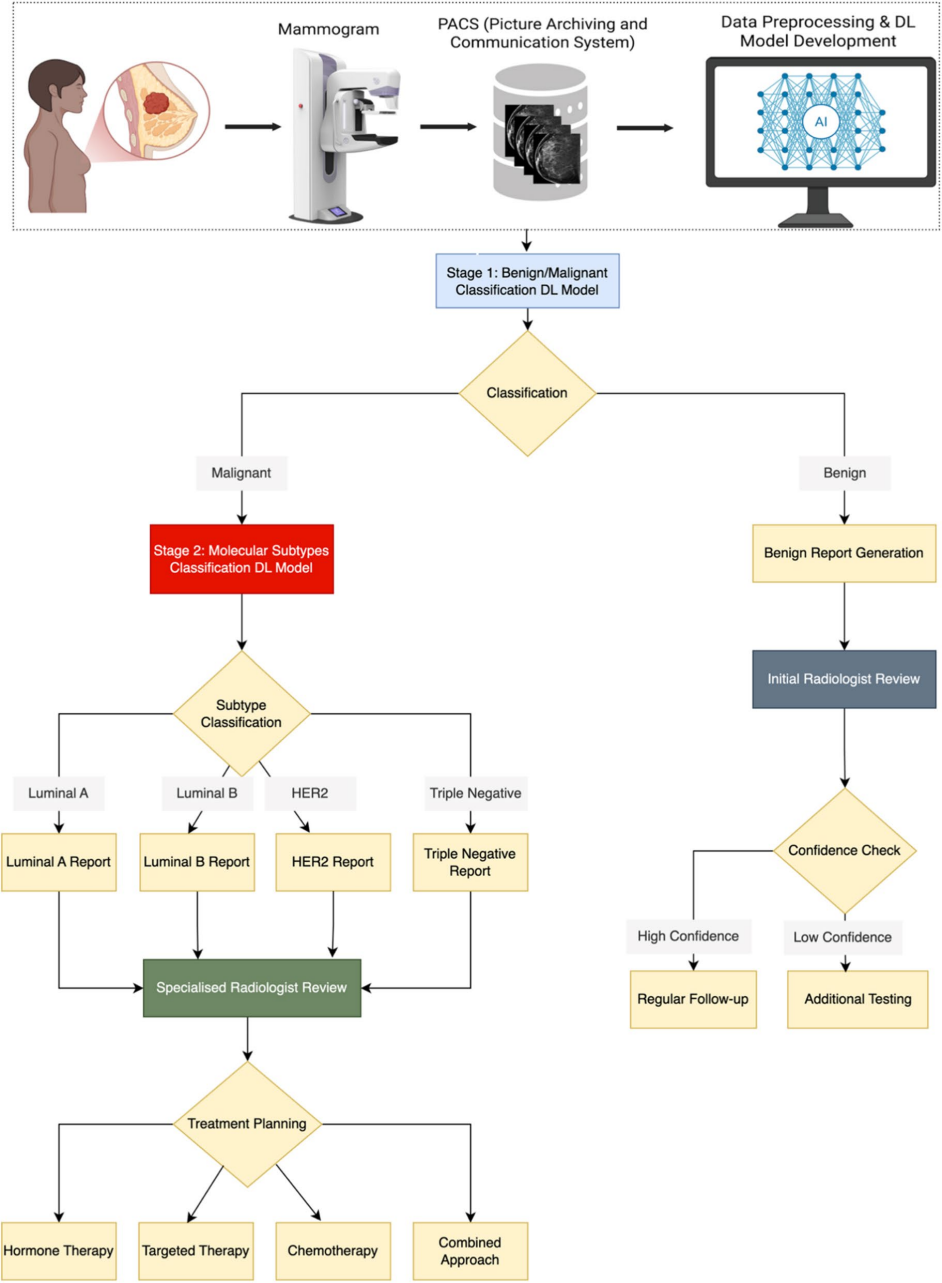
A flowchart illustrating a two-stage DL workflow for breast cancer diagnosis and treatment planning from mammograms. The system first classifies lesions as benign or malignant, then further categorizes malignant cases into molecular subtypes to inform therapeutic strategies

**Fig. 3:**
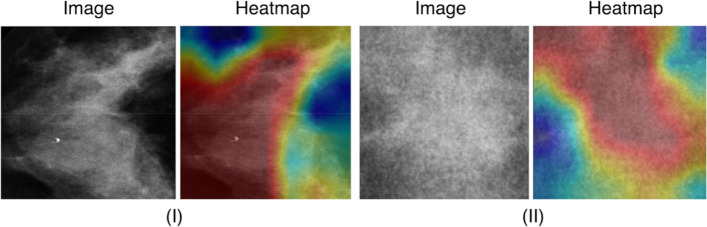
Grad-CAM visualization of tumor masses. The figure shows two tumor mass examples, I and II, with their corresponding Grad-CAM heatmaps. The red areas highlight the most important regions the network focused on, while blue and yellow areas indicate less significant regions

**Fig. 4:**
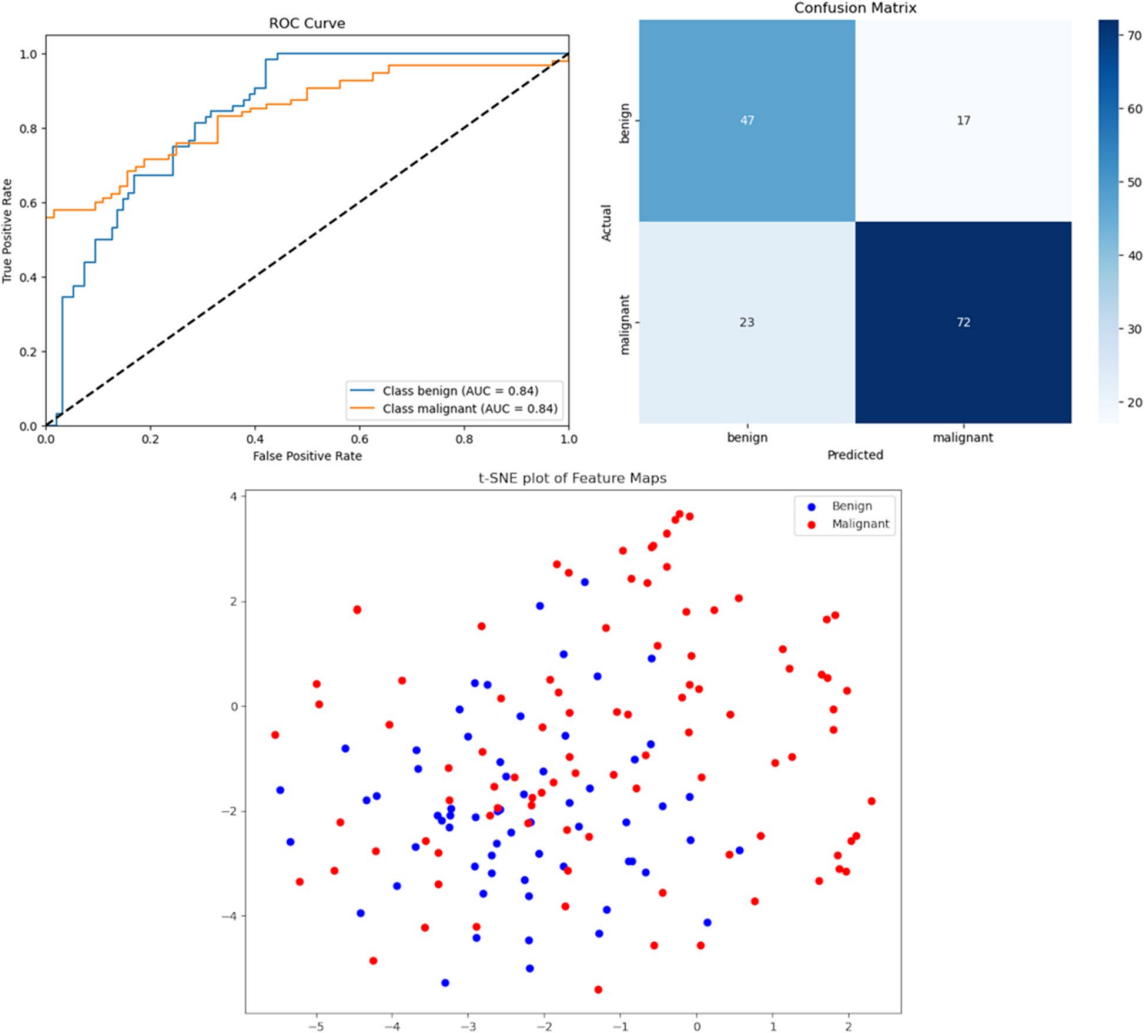
AUROC and confusion matrix. These plots show how well the model predicted whether a tumor is benign or malignant. The AUROC measures overall performance, while the confusion matrix shows the number of correct and incorrect predictions. Additionally, a t-SNE plot of the extracted feature maps demonstrates how the model clusters similar tumor masses in latent space, with each color representing a different class (benign or malignant)

The clinical context for using this workflow is presented in Fig. 2 and Table 1. It combined YOLOv9 and ResNet-50 models with Grad-CAM (Fig. 3) and t-SNE explainability, trained on widely available mammograms, to predict breast cancer molecular subtypes (Luminal A/B, HER2-enriched and Triple-Negative). The classification of benign versus malignant cases achieved an accuracy, precision, and recall of 74.84%, 73.14% and 67.14%, respectively (Fig. 4). The prediction of molecular subtypes achieved an accuracy of 58% on limited test samples.

**Table 1:** Benefits of AI for radiological image analysis

Benefits for radiologists	Benefits for patients
1. Clinical decision support	1. Faster diagnosis
• Assists in rapid preliminary classification • Provides probability scores for each molecular subtype • Highlights suspicious regions through heatmaps	• Reduces the waiting time for initial assessments • Enables earlier treatment planning • Streamlines the diagnostic process
2. Workflow enhancement	2. Improved accuracy
• Reduces time spent on initial assessments • Serves as a ‘second opinion’ tool • Standardizes the reporting process	• Combines human expertise with AI assistance • Reduces chances of misclassification
3. Educational value	3. Fewer biopsies
• Visualization tools help understand feature importance • Could be used for training radiologists • Provides objective feedback (e.g., second opinion)	• High confidence predictions could reduce number of required biopsies

### Session 4: deep learning-based histopathology analysis reveals tissue heterogeneity in breast cancer: a pipeline from annotation to interpretability

Presenters: Oscar Maiques and Lisa Schneider.

#### Data sources and preprocessing

**Fig. 5:**
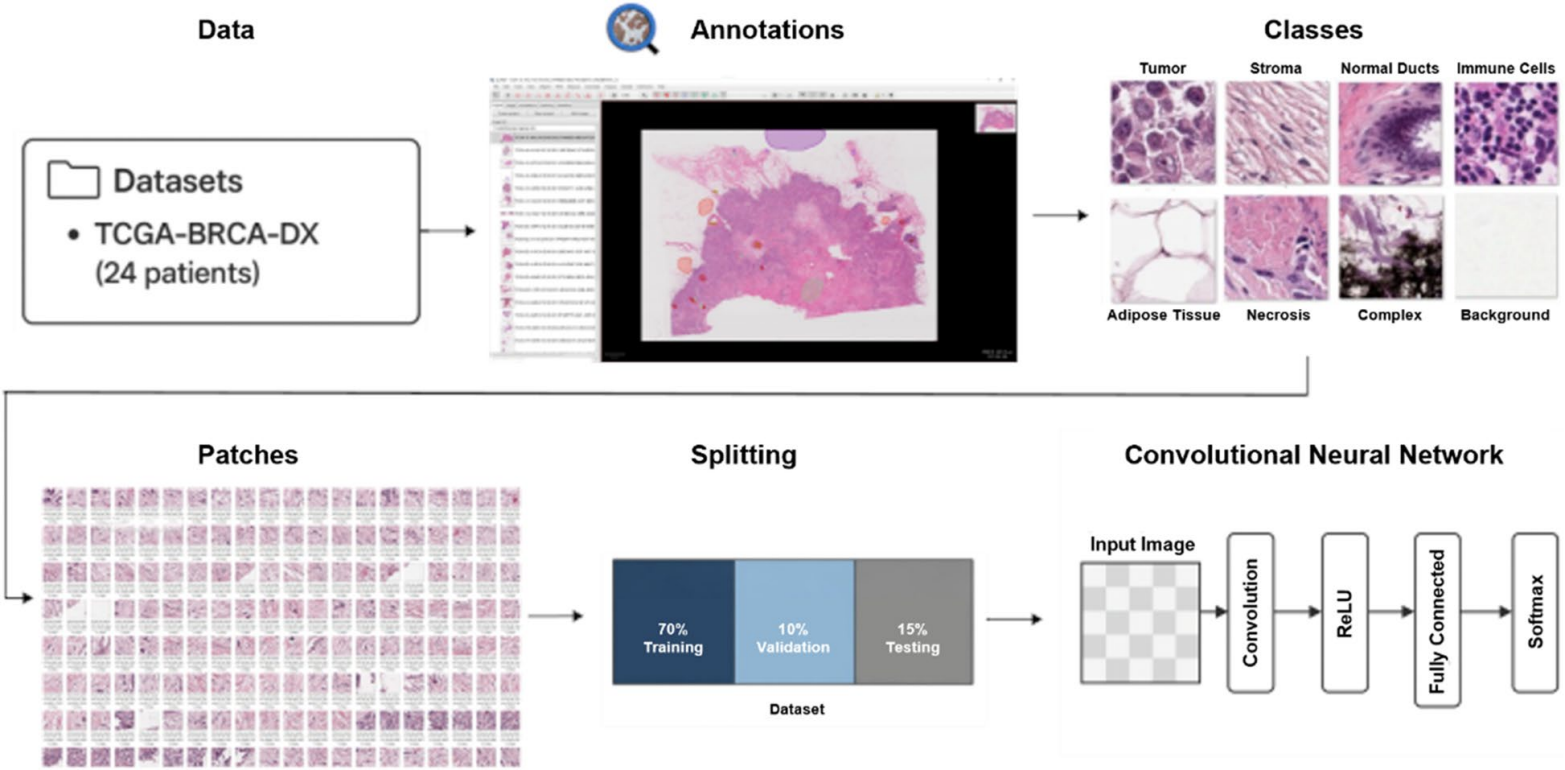
Overview of the DL pipeline for histopathological image classification. WSIs from the TCGA-BRCA dataset (24 patients) were annotated using QuPath to identify eight histological classes: Tumor, Stroma, Normal Ducts, Immune Cells, Adipose Tissue, Necrosis, Complex and Background. Annotated regions were extracted as fixed-size image patches. These patches were then split into training (70%), validation (10%) and testing (15%) sets. A CNN was trained on the image patches to classify tissue regions based on the annotated histological classes. The CNN architecture includes convolutional layers, ReLU activation, fully connected layers and a final Softmax layer for multiclass classification

For this session, we used Whole Slide Images (WSIs) from the TCGA-BRCA dataset, aiming to classify tissue regions into eight distinct histological classes: Tumor, Stroma, Normal Ducts, Immune Cells, Adipose, Necrosis, Complex and Background (Fig. 5).

The analysis began with the careful annotation of WSIs using QuPath [30]. Expert-driven annotations delineated key histological areas, forming the foundation for subsequent analysis. To facilitate training, the annotated WSIs were divided into 256 × 256 pixel patches. Each patch was labelled with its corresponding class, ensuring that no patient’s data were mixed across training, validation and testing datasets.

#### Methods

**Fig. 6:**
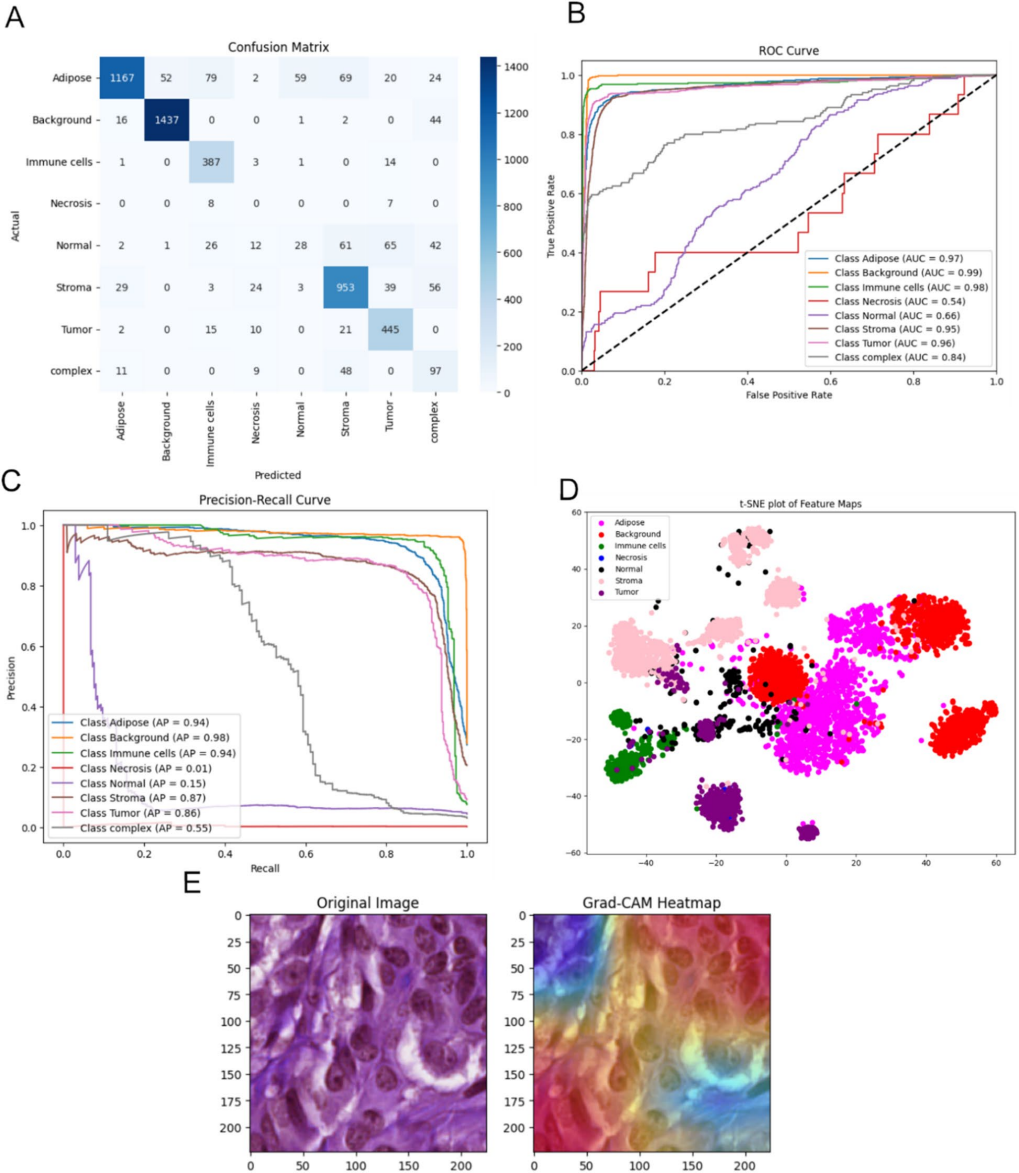
Evaluation and interpretability of the histopathology image classification model. Model's performance across eight histological classes: Adipose, Background, Immune cells, Necrosis, Normal, Stroma, Tumor and Complex. **A** Corresponding confusion matrix. **B** ROC curves, with AUC reported for each class. **C** Precision-recall curves. **D** t-SNE plot of the extracted feature maps demonstrates how the model clusters similar image patches in latent space, with each color representing a different histological class. **E** Histological image is shown on the left, while the corresponding Grad-CAM heatmap in the center reveals the spatial regions the model attends to when making predictions

The pipeline was built around a ResNet-50 model, a deep convolutional neural network (CNN) renowned for its capacity to learn complex visual features. The model's performance was evaluated on the held-out test set. Metrics such as accuracy, precision, recall and F1-score provided a comprehensive picture of its classification ability. Visual tools, including confusion matrices and receiver operating characteristic (ROC) curves, highlighted the model's performance across classes (Fig. 6).

The annotated and labelled patches were processed through a comprehensive digital pathology pipeline. Each step of the pipeline contributed to refining our understanding of tissue composition and heterogeneity. The process began with data augmentation, where techniques such as rotation and flipping were applied to the dataset. These transformations introduced variability that mimicked real-world scenarios, allowing the model to better generalize, particularly for classes that were underrepresented. For model training, we utilized a ResNet-50 architecture, implemented through PyTorch libraries [31]. The model was optimized using the Adam optimizer [32] and trained with cross-entropy loss, a combination well-suited for multiclass classification tasks. Iterative validation played a crucial role, ensuring the model converged effectively while minimizing the risk of overfitting.

To assess and interpret the results, we incorporated visualization and explainability techniques into the pipeline. t-SNE plots provided a visual representation of how well the model separated classes in high-dimensional feature spaces. These plots revealed clusters corresponding to different classes, offering valuable insights into the separability and distinctiveness of each tissue type. Additionally, we employed Grad-CAM heatmaps to delve deeper into the model’s decision-making process. These heatmaps highlighted the specific regions of each patch that the model deemed most important for classification, ensuring the network's focus aligned with known histopathological patterns. Finally, we turned our attention to quantification and overlay. At the slide level, we quantified the proportion of each class, offering a detailed overview of the tissue composition in each sample. At the patch level, class-specific overlays were superimposed on the WSIs, creating spatially resolved maps that visually captured the distribution of classified regions. These maps provided an intuitive way to validate the model’s predictions and improved the explainability of our results.

#### Results

The model provided a robust starting point for feature extraction and classification, effectively capturing histological patterns in the data. However, while our model demonstrated great capabilities to distinguish tumor tissues from other classes, the simplicity of labelling tumor regions under a single Tumor class might have limited the model’s ability to capture the full spectrum of biological morphology and variability among different tumors, since it was not relevant to the task. Notably, breast cancer tumors can exhibit vast histological diversity depending on their subtype, grade and morphological features, which is highly relevant to consider in future annotations or pipelines.

To gain insights on more intricate histological patterns in tumor tissue, we compared the ResNet-50 model with a simple linear classifier added on top of a Bayer in-house foundation model built with cutting-edge architectures such as vision transformer (ViT) [33]. Foundation models are typically not trained on a specific task but independently acquire knowledge from vast amount of data – here 64 M patches – in a so-called self-supervised manner. This allows the model to explore and uncover – eventually even unknown – patterns independently, and therefore, potentially better capture the biological morphology and variability among different tumors. To investigate the potential of this, we incorporated visualization techniques such as t-SNE plots into the pipeline. These plots provided a lens into the heterogeneity within the tumor class, revealing distinct clusters that aligned with morphological differences. This allowed us to interrogate these clusters further, offering deeper insights into tumor heterogeneity and its potential biological implications. This state-of-the-art approach significantly expanded the potential for feature extraction and predictive accuracy, offering a glimpse into future possibilities for our pipeline.

### Session 5: lung cancer OMOP data analysis

Presenters: Eng Hooi (Cheryl) Tan and Bertrand De Meulder.

#### Data sources and preprocessing

In the OPTIMA project, we have established a network of clinical sites having medical data on breast, lung and prostate cancer. We used lung data mapped to the OMOP CDM to facilitate the replication of our analyses [34]. To phenotype a lung cancer cohort, we followed a workflow using distributed analytics [35]. We generated codelists – also known as concept sets – for lung cancer, including broad and narrow definitions, and small cell lung cancer (SCLC). The concept sets were generated across 11 data sources from 8 countries (Belgium, Estonia, Germany, Italy, Romania, Spain, UK and US), including primary care databases (CPRD GOLD [36], SIDIAP [37], THIN [38], IQVIA DA Germany), cancer databases (IQVIA OncoEMR) and biobanks (Estonian Biobank). The concept sets were implemented as cohort diagnostics using the PhenotypeR package developed in the OPTIMA project. The diagnostics included cohort counts and overlap, concept counts, index event breakdown, orphan concepts, age and sex distribution, incidence and prevalence and large-scale characterization. A lung oncologist reviewed the diagnostics.

The data used in this session are available for OPTIMA researchers in a hybrid central/federated network setting. The OPTIMA central data platform hosted by Helmholtz-Zentrum Dresden-Rossendorf (HZDR) provides access to the importable data. The federated data remain on data owners’ secure servers, and the OPTIMA data partners are provided the analytical code to run locally, sharing only aggregated results. Both central and federated results are then compiled and presented on the OPTIMA central data platform.

#### Methods

**Fig. 7:**
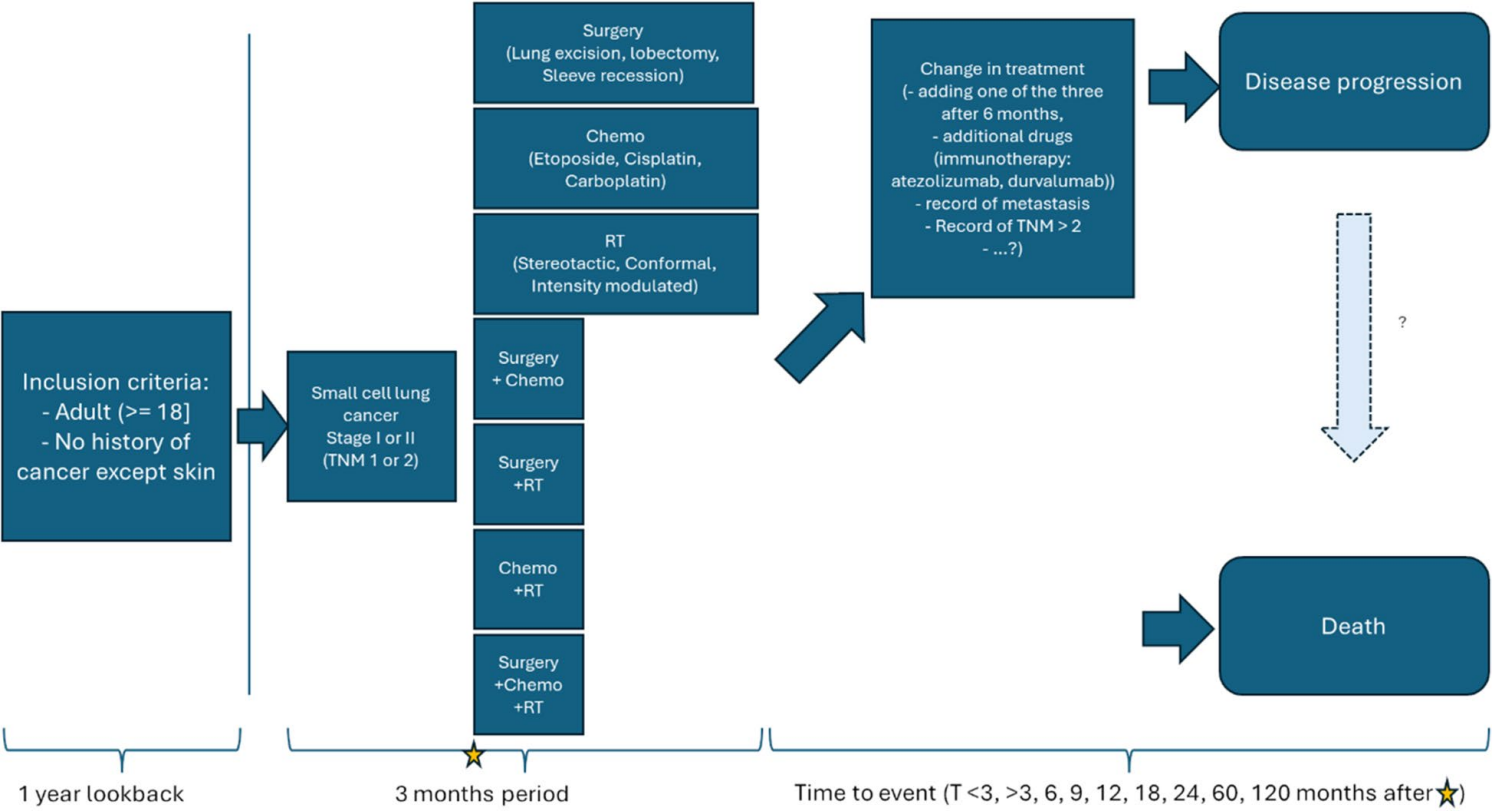
Summary of the lung cancer patient cohorts. The question marks reflect unknowns in the analysis plan that will be answered iteratively; the list of relevant features to consider for disease progression is not final, and it is unclear whether we will be able to codify the step between disease progression and death

The first step in analyzing OMOP data is to design a study protocol to address the research questions and to define the patient cohorts of interest. Hence, we focused on limited-stage SCLC patients, the different treatments administered and clinical outcomes. We used information from the cohort diagnostics as a basis for defining the cohorts. Together with lung cancer clinicians, we defined the cohorts as the following: all participants are required to be at least 18 years old and have at least one year of data availability prior to diagnosis (look-back period). To be included in the SCLC cohort, patients must have no history of cancer except for skin cancer, a record of SCLC diagnosis and staging information (T stage I or II in TNM staging) recorded within a month prior and 2 months after SCLC to allow for diagnosis and record time in the clinic. We identified the different treatments used in limited-stage SCLC according to the National Comprehensive Cancer Network (NCCN) 2025 clinical guidelines [39]. Depending on tumor characteristics, treatment options are surgery (lung excision, lobectomy or sleeve recession), chemotherapy (Carboplatin, Cisplatin, Etoposide), radiotherapy (stereotactic, conformal, intensity modulated) or combinations of the three options. We used either disease progression or death as clinical endpoints. We used the OMOP definition of a progressive disease cohort, which covers established progression criteria such as the Response Evaluation Criteria in Solid Tumors (RECIST) [40] criteria for SCLC, and defined several time points to check for disease progression in the first 3 months, after 3 months, within 6, 9, 12, 18 and 24 months, within 5 years or after 10 years (Fig. 7). All cohorts were defined using the OHDSI ATLAS software [41] available in the OPTIMA central analytics platform. The cohorts were then packaged in an R Shiny app [42] adapted from the OHDSI CohortDiagnostics [43]. We benefited greatly from the previous work done by PIONEER, PIONEER + and UroEvidenceHub initiatives in developing the methods used in this session.

#### Results

From the diagnostics review, the lung cancer codes included were in line with clinical expectations, as were the age and sex distribution of lung cancer cases across databases. The incidence rates were checked with global estimates, and discrepancies were investigated.

The analysis revealed large differences between databases, mainly in the underlying features that are effectively measured for the patients included in the database. For example, TNM stage can be recorded either as a combined score, individual T, N and M score, using clinical (c prefix) or histopathological (p prefix) data, translated into staging information or completely unavailable either due to oversight in the OMOP mapping process or from being absent from the source database. Moreover, the analysis also confirmed that limited-stage SCLC is a relatively rare disease. Out of all lung cancer cases, SCLC amounts for around 15% of the cases, and early-stage SCLC (stage I or II) is around 5% of those, consistent with the literature [44, 45]. This greatly reduced the number of patient records available for our analysis. Across the OPTIMA data network, we expect to be able to retrieve information for around 7,000 patients, which is a large number compared to state-of-the-art literature [46, 47].

## Discussion

Our integrated analysis of multiple data modalities provided significant insights into the four core research questions posed by the OPTIMA consortium.

### Q1: genomic profiling and treatment outcomes

The multi-omics workflow in Session 2 demonstrated the potential of genomic profiling to inform therapeutic decisions in both primary and metastatic settings. Future expansions of this workflow include exploring how pharmacogenomics could be used to inform pharmacovigilance. Predicting adverse reactions to chemotherapeutic agents in conjunction with identifying treatments that a patient is likely to be susceptible to minimizes unnecessary toxicity and avoids potentially ineffective treatments for patients.

### Q2: tumor heterogeneity and treatment response

Intratumoral heterogeneity is associated with resistance to treatment and breast cancer recurrence and metastasis, and it is a crucial factor to consider in research and clinical management [48]. The digital pathology workflow in Session 4 together with the transcriptomics workflow in Session 2 revealed distinct patterns of tissue composition across molecular subtypes. Combining both would be key to unravel tumor heterogeneity and evolution.

The DL model used in the digital pathology workflow successfully classified eight distinct histological components, providing quantitative measures of tumor heterogeneity. While our pipeline laid the groundwork with a classical ResNet-50 approach, the extension with Bayer's foundation model highlighted the opportunities for further innovation. By embedding visualization and explainability techniques, our pipeline not only achieved accurate classification but also provided a framework to explore and understand tissue heterogeneity, driving progress towards more personalized and effective cancer treatments.

Future work will focus on refining tumor subclassification, integrating multimodal data and leveraging state-of-the-art AI architectures to enhance the resolution and depth of histological insights. Future expansions of the transcriptomics workflow will include refining the workflow and applying it to other OPTIMA datasets such as targeted RNAseq data from the PALOMA-3 trial [49] and integration with digital pathology to gain a comprehensive understanding of the complex interplay between tumor heterogeneity and clinical outcomes in breast cancer.

### Q3: non-invasive tumor assessment technologies

A large portion of the information found within EHRs is stored in the form of unstructured free text and images. AI methods are leveraged to extract this information and harness their potential for improving patient care and outcome.

The DL-based radiological analysis in Session 3 showed promising results for non-invasive tumor assessment. Performance metrics confirmed that our model achieves a diagnostic and predictive performance comparable to or exceeding standard methods, with high specificity and sensitivity. Predicting molecular subtypes directly from mammograms provides radiologists with vital information for clinical decision-making. Model explainability was enhanced using Grad-CAM and t-SNE visualization techniques, building trust in the technology and providing actionable insights for treatment planning, further reducing the need for biopsies. This methodology addresses the limitations of traditional biopsies, including patient discomfort, procedural risks and resource demands. Replacing these invasive procedures with non-invasive, AI-enhanced imaging improves patient experience and could offer a scalable and cost-effective diagnostic solution. The pipeline’s scalability also suggests its potential application to other imaging modalities. While these results demonstrate potential, they also highlight areas for improvement such as training with larger datasets.

Analysis of free text radiology reports in Session 1 showcased the feasibility of using NLP techniques to automatically extract oncology-related information from these rich reports that could improve monitoring of breast cancer patients. Future directions will include capturing breast cancer-related information other than metastasis site (e.g., tumor characteristics), integration with other workflows (e.g., AI radiological image analysis) and application of NLP techniques such as large language models (LLMs).

In the multi-omics workflow, future expansions will include analysis of ctDNA from breast cancer patients to demonstrate the benefits of longitudinal samples, providing a dynamic view of tumor progression such as clonal evolution and the effectiveness of current treatments.

### Q4: stage I-II SCLC analysis

The lung cancer phenotyping workflow is an ongoing study and will be part of a subsequent larger OPTIMA study, characterizing all stages of SCLC and studying treatment patterns and time to event on several clinically relevant endpoints. We are also exploring the possibility of follow-up studies such as comparing treatment choices and clinical endpoints across the OPTIMA clinical sites and other hospitals, knowledge transfer or clinical guideline updates, comparing the results of this study to those of published randomized control trials and expanding the study once validated through the European Respiratory Society to enroll new data partners and augment the number of patient records included. Moreover, we will be exploring the possibility of combining OMOP-based analyses with ML/AI analyses. We consider OMOP data analysis as a first step to create cohorts or groups of patients with distinct clinical features or outcomes of interest, which could then feed into AI workflows to perform predictive analysis, or include other data sources such as imaging and/or molecular measurements, towards multimodal analysis.

## Conclusions

The OPTIMA workshop demonstrated the potential of multimodal AI-driven analysis in advancing breast and lung cancer research while highlighting important areas for future development. Our findings make several contributions to the field: first, the combination of genomic profiling with clinical outcomes data provides a framework for more personalized treatment selection in breast cancer; second, the ability to quantify and map tissue composition and immune cell infiltration patterns offers new possibilities for treatment stratification and monitoring; third, progress in non-invasive assessment methods through AI-enhanced imaging outlines a path towards reducing reliance on invasive procedures while maintaining diagnostic accuracy; fourth, using NLP to extract information from EHR could improve and accelerate non-invasive patient monitoring; fifth, OMOP-based analysis could be used as a first step in more complex workflows towards multimodal modelling or analysis, harnessing the power of both large population-based clinical datasets and deeper, more focused imaging or omics datasets. These achievements align directly with the OPTIMA consortium's mission to optimize cancer treatment through AI.

The workflows developed during this workshop provide a foundation for continued collaborative research within the OPTIMA consortium. However, significant work remains to translate these findings into clinical practice. Future efforts will focus on getting access to well-annotated cohorts with multimodal data, including clinical, genomic, imaging and pathology information. Furthermore, technical challenges persist in unlocking and linking diverse data types while maintaining data privacy and security. As we move forward, focusing on clinical applicability while advancing technical capabilities will be crucial for realizing the full potential of AI-driven approaches in cancer management.

## Data Availability

The data that support the findings of this workshop are available from the OPTIMA consortium.

## Abbreviations

OPTIMA: Optimal Treatment for Patients with Solid Tumours in Europe Through Artificial Intelligence
EU: European Union
NSCLC: Non-small cell lung cancer
SCLC: Small cell lung cancer
AI: Artificial Intelligence
EHRs: Electronic health records
BCNB: Breast Cancer Now Biobank
BCI: Barts Cancer Institute
OMOP: Observational Medical Outcome Partnership
CDM: Common data model
BH: Barts Health NHS Trust
NLP: Natural Language Processing
TCGA-BRCA: The Cancer Genome Atlas Breast Invasive Carcinoma
MAF: Mutation Annotation Format
ER: Estrogen receptor
HER2: Human epidermal growth factor receptor 2
TCIA: The Cancer Imaging Archive
CMMD: Chinese Mammography Database
CC: Craniocaudal
MLO: Mediolateral oblique
DL: Deep learning
YOLOv9: You Only Look Once version 9
ROIs: Regions of interest
SGD: Stochastic Gradient Descent
AUROC: Area Under the Receiver Operating Characteristic curve
Grad-CAM: Gradient-weighted Class Activation Mapping
t-SNE: T-Distributed Stochastic Neighbor Embedding
WSIs: Whole slide images
CNN: Convolutional neural network
ROC: Receiver Operating Characteristic
ViT: Vision Transformer
HZDR: Helmholtz-Zentrum Dresden-Rossendorf
NCCN: National Comprehensive Cancer Network
RECIST: Response Evaluation Criteria in Solid Tumors
LLMs: Large language models
